# Adoptive T-cell immunotherapy from third-party donors: characterization of donors and set up of a T-cell donor registry

**DOI:** 10.3389/fimmu.2012.00410

**Published:** 2013-01-28

**Authors:** Britta Eiz-Vesper, Britta Maecker-Kolhoff, Rainer Blasczyk

**Affiliations:** ^1^Institute for Transfusion Medicine, Hannover Medical SchoolHannover, Germany; ^2^Department of Pediatric Hematology and Oncology, Hannover Medical SchoolHannover, Germany

**Keywords:** adoptive immunotherapy, T-cell therapy, antiviral T lymphocytes, cytomegalovirus, Epstein–Barr virus, adenovirus

## Abstract

Infection with and reactivation of human cytomegalovirus (CMV), Epstein-Barr virus (EBV), and adenovirus (ADV) are frequent and severe complications in immunocompromised recipients after hematopoietic stem cell transplantation (HSCT) or solid organ transplantation (SOT). These serious adverse events are associated with significant morbidity and mortality. Donor lymphocyte infusions (DLIs) are often used to treat both viral infections and leukemia relapses after transplantation but are associated with potentially life-threatening graft-versus-host disease (GvHD). Adoptive immunotherapy with virus-specific cytotoxic effector T cells (CTLs) derived from seropositive donors can rapidly reconstitute antiviral immunity after HSCT and organ transplantation. Therefore, it can effectively prevent the clinical manifestation of these viruses with no significant acute toxicity or increased risk of GvHD. In conditions, where patients receiving an allogeneic cord blood (CB) transplant or a transplant from a virus-seronegative donor and since donor blood is generally not available for solid organ recipients, allogeneic third party T-cell donors would offer an alternative option. Recent studies showed that during granulocyte colony-stimulating factor (G-CSF) mobilization, the functional activity of antiviral memory T cells is impaired for a long period. This finding suggests that even stem cell donors may not be the best source of T cells. Under these circumstances, partially human leukocyte antigen (HLA)-matched virus-specific CTLs from healthy seropositive individuals may be a promising option. Therefore, frequency assessments of virus-specific memory T cells in HLA-typed healthy donors as well as in HSCT/SOT donors using a high throughput T-cell assay were performed over a period of 4 years at Hannover Medical School. This chapter will address the relevance and potential of a third-party T-cell donor registry and will discuss its clinical implication for adoptive T-cell immunotherapy.

## Introduction

Hematopoietic stem cell transplantation (HSCT) is used to cure many malignant, benign and genetic disorders of the bone marrow, solid tumors, immunodeficiencies, metabolic, and autoimmune disorders (Ljungman et al., 2010). HSCT is generally performed after administration of sublethal doses of chemotherapy or chemoradiotherapy to achieve myeloablation, immunosuppression and eradication of abnormal cells. Intensive immunosuppressive therapy for prevention of graft rejection and graft-versus-host disease (GvHD) and for treatment of GvHD puts the patients at risk of opportunistic infections due to an ablated or severely compromised T-cell immune response. Such invasive conditioning procedures lead to a lack of immunological competence, which results mainly in a decrease in the number of CD3+ T lymphocytes in the patient's peripheral blood. Lymphopenia increases the patient's risk of *de novo* infection or reactivation of a latent virus. This mainly occurs during the early post-transplantation period and usually leads to a disseminated disease. The immune reconstitution period following HSCT (as long as 3–6 months) is therefore accompanied by a high incidence of infections with various pathogens that are normally controlled by T-cell immunity.

## Role of T cells in transplantation

In allogeneic HSCT, the presence of a defined number of donor-derived T cells in the stem cell graft may prevent graft failures, infections or reactions caused by different pathogens (graft-versus-infection effect, GvI) as well as disease relapses (graft-versus-leukemia/graft-versus-tumor effect, GvL/GvT). On the other hand, an excessive number of T cells may increase the risk of developing GvHD. Major complications of stem cell and organ transplantation, such as graft rejection and GvHD, are countered by suppressing the host immune system via chemotherapy and radiation, immunosuppressive drugs, or conditioning regimens such as *in vivo* or *in vitro* T-cell depletion (Gooley et al., 2010). While immunocompromised, the patient is rendered susceptible to a number of viral infections mainly caused by endogenous herpes viruses like cytomegalovirus (CMV) and Epstein-Barr virus (EBV) and by lytic agents such as adenovirus (ADV). Infections by several other viruses such as polyoma virus BK (BKV) and human herpesvirus 6 (HHV-6) as well as by invasive fungal pathogens such as *Aspergillus* are also reported to cause significant complications after stem cell and solid organ transplantation (SOT) (Marr et al., 2002; Garcia-Vidal et al., 2008; Pappas et al., 2010; Amir et al., 2011; Breuer et al., 2012).

## Viral complications after allogeneic stem cell transplantation and organ transplantation

### CMV infection

Human CMV is a persistent β-herpesvirus that infects most healthy individuals during the first years of life (Khan, 2007). Healthy CMV-seropositive individuals have a high number of CMV-specific CD8+ and CD4+ T lymphocytes, which are essential to control viral reactivation without clinical symptoms (Rauser et al., 2004). Immunocompromised CMV-seropositive patients (R+) receiving a graft from a seronegative donor (D−) have a high risk of CMV disease (Zhou et al., 2009; Borchers et al., 2011; Ugarte-Torres et al., 2011). Additionally, it was reported that CMV reactivation developed in 96% of D+R+ patients but in less than 50% of D+R– patients (Lilleri et al., 2008, 2012). Reactivation of CMV results in significant morbidity and mortality; clinical manifestations include interstitial pneumonitis, gastroenteritis, fever, hepatitis, encephalitis, and retinitis (Einsele et al., 2008; Fujita et al., 2008a,b). While ganciclovir, valganciclovir, foscarnet, and cidofovir may help to prevent or treat CMV infection, they are associated with late-onset disease and serious side-effects, such as nephrotoxicity, myelosuppression, and impaired immune reconstitution, leading to an increase in invasive fungal infections and bacterial sepsis (Broers et al., 2000; Battiwalla et al., 2007; Fujita et al., 2008a,b; Boeckh and Ljungman, 2009). Furthermore, these drugs are often ineffective due to primary or secondary resistance, and patients still develop CMV disease in spite of antiviral treatment (Einsele et al., 2000; Mori et al., 2000; Fuji et al., 2011). Hence, cellular immunity is important for the control of CMV infection, and CMV-specific CD8+ and CD4+ T cells are essential for efficient immune protection after both primary activation and reactivation of latent CMV disease (Fujita et al., 2008a,b; Feuchtinger et al., 2010; Fuji et al., 2011).

### EBV infection

EBV-associated post-transplant lymphoproliferative disease (PTLD) occurring after HSCT or SOT is a potentially life-threatening condition (Cohen, 2000; Gottschalk et al., 2005). The overall incidence of PTLD after allogeneic HSCT is less than 1%, but was reported to be increased after transplantation with human leukocyte antigen (HLA)-mismatched or T-cell-depleted grafts (Landgren et al., 2009). Further risk factors for the development of PTLD include the degree and duration of immunosuppressive treatment and the use of antithymocyte globulin (ATG) with reduced-intensity transplant conditioning (Landgren et al., 2009; Pidala et al., 2011). PTLD developing after hematopoietic SCT usually results from donor B cells and appears within the first 6–12 months post-transplant, when profound deficiencies of EBV-specific cytotoxic effector T cells (CTLs) (EBV-CTLs) occur (Meij et al., 2003). In SOT, the incidence varies with the type of organ (1–2% after kidney transplantation and up to 20% after thoracic organ transplantation) (Trappe et al., 2012). Eighty five percentage of pediatric PTLDs and 60–70% of adult PTLDs in Europe are EBV-associated. Insufficient EBV-specific T-cell responses have been linked to a higher risk of PTLD development (Guppy et al., 2007). PTLD in SOT recipients usually originates from recipient B cells; however, a significant percentage (10–15%) of predominantly early PTLD in kidney or liver graft recipients restricted to the organ graft displays donor origin (Olagne et al., 2011). Treatment includes reduction of immunosuppressive drugs as far as tolerated, immunotherapy (monoclonal antibodies like Rituximab), and cytotoxic chemotherapy. Preemptive therapy with CD20 monoclonal antibodies (Rituximab) has been attempted and may control EBV-associated lymphoproliferation (Kuehnle et al., 2000; van Esser et al., 2002; Trappe et al., 2012). Treatment is often complicated by side effects, and severe complications are foreseeable in patients with pre-existing organ dysfunction. Second line treatment options are scarce and have not been tested in clinical studies. Adoptive T-cell therapy using EBV-CTLs has been successfully employed for prophylaxis and treatment of PTLD in high-risk patients (Haque et al., 2007; Heslop et al., 2010; Shen et al., 2011).

### ADV infection

The incidence of ADV infection ranges from 3 to 20%, and is significantly higher in pediatric patients (Feuchtinger et al., 2005; Fowler et al., 2010). Overall ADV-associated mortality ranges from 18 to 26%. ADV infection may involve the respiratory, gastrointestinal and/or urinary tract. The diagnosis of ADV infection can be difficult due to its complexity. The most common cause of adenoviral infection after HSCT is reactivation of a latent virus persisting, for example, in intestinal mucosa. Furthermore, there are reports indicating a more than 4-fold increased risk of ADV infection in patients with grafts from seropositive donors (Runde et al., 2001; Walls et al., 2003; Fowler et al., 2010). Risk factors underlying this increase are: T cell-depleted grafts (Chakrabarti et al., 2002; Lion et al., 2003), allogeneic graft from matched unrelated donors (MUD) (Ebner et al., 2005), acute GvHD (Bruno et al., 2003), cytotoxic and immunosuppressive therapy (Watcharananan et al., 2010), and lymphocytopenia (Feuchtinger et al., 2008; Watcharananan et al., 2010). Currently, 53 different human serotypes (ADV1 to ADV53) are classified into seven species (A to G). The most prevalent serotypes in transplant patients are ADV 1, 2, 5, 31, and 41. In 49 pediatric patients who received a stem cell transplant at Hannover Medical School (MHH) from 2003 to 2011, sequence analysis revealed ADV species A (1% ADV18, 20.5% ADV31), B (4% ADV3), C (20.5% ADV1, 32% ADV2, 4% ADV6), E (8% ADV4), and F (10% ADV41) (Mynarek et al., submitted). Incidence of ADV viremia was high (50%) with mostly asymptomatic patients, who developed only low viral loads. Despite a low ADV-related mortality rate of 0.84% in this cohort, high peak ADV blood loads were a significant and independent risk factor for survival after HSCT. Cidofovir and Ribavirin have been used to treat immunocompromised patients suffering from ADV infection (Lankester et al., 2004; Lindemans et al., 2010). However, these antiviral agents were shown to limit but not clear the infection and are associated with severe side effects. Recent studies indicate that the elimination of ADV is only achieved by recovery of cellular immunity (Feuchtinger et al., 2006). Therefore, the adoptive transfer of antigen-specific T cells could be an effective and non-toxic alternative strategy.

## Adoptive T-cell therapy using antiviral T cells

Although donor lymphocyte infusions (DLIs) can be used after transplantation to treat both viral infections and leukemia relapses, they are associated with potentially life-threatening GvHD (Collins et al., 2000; Choi et al., 2005). The shortcomings of conventional therapies have increased the interest in an immunotherapeutic approach to treat viral disorders. It was recently shown that the adoptive transfer of antiviral cytotoxic T lymphocytes directed against CMV (Einsele et al., 2008; Mackinnon et al., 2008; Brestrich et al., 2009; Feuchtinger et al., 2010; Peggs et al., 2011), EBV (Haque et al., 2010; Heslop et al., 2010; Moosmann et al., 2010; Doubrovina et al., 2012), and ADV (Feuchtinger et al., 2008; Hoffman, 2009; Zandvliet et al., 2010; Qasim et al., 2011) isolated from seropositive donors can rapidly reconstitute antiviral immunity after stem cell and organ transplantation without significant toxicity and with limited increase in GvHD. Infusions of peripheral blood-derived T-lymphocyte lines enriched in multivirus (CMV, EBV, and ADV)-specific T cells reproducibly controlled infections by all three viruses after allogeneic HSCT and may form the basis of future adoptive immunotherapy trials in patients at risk of multiple infections (Leen et al., 2006; Fujita et al., 2008a,b; Khanna et al., 2011; Zandvliet et al., 2011; Gerdemann et al., 2012).

Although the minimal frequency of antigen-specific T cells required to mediate an antiviral effect in patients is not known it is likely to vary widely depending on the target antigen and other factors, including quantitative and even more qualitative properties of the effector T cells as well as the host environment. The importance of the host environment to facilitate persistence and function of transferred T cells has recently been elucidated (Berger et al., 2009).

### Adoptive T-cell therapy for CMV infection

The presence of CD8+ and CD4+ antiviral T cells was reported to be essential in controlling viral infection and reactivation by restoring cellular immunity. Since the first promising results began to emerge in the early 1990s (Greenberg et al., 1991; Riddell et al., 1991), different strategies to generate virus-specific T lymphocytes for clinical use have been described. In 1995, Walter and colleagues demonstrated that CMV reactivation after HLA-identical allogeneic HSCT can be prevented by adoptive transfer of CMV-specific cytotoxic T cells, which were generated *in vitro* from the transplant donor and transferred to the patient (Walter et al., 1995). To be suitable for clinical applications, the cells used for adoptive T-cell transfer must be virus-specific T cells generated by *in vitro* induction and expansion from a small number of precursor cells, over a short period of culture, under highly reproducible conditions, and in accordance with good manufacturing practice (GMP). CMV-specific memory T cells are present at high frequencies in the blood of healthy CMV-seropositive donors. Typically, they represent 0.5% to 4% of the CD8+ T-cell pool and 0.05% to 1.6% of the CD4+ T helper (Th) cell pool (Rentenaar et al., 2000; Cwynarski et al., 2001). Most protocols for the generation of virus-specific T cells use peptide-loaded monocyte-derived dendritic cells (DCs), artificial antigen-presenting cells (aAPCs), or CMV-infected immature dendritic cells as stimulator cells (Sun et al., 1999; Peggs et al., 2001; Carlsson et al., 2003; Oelke et al., 2003; Lozza et al., 2005; Paine et al., 2007, 2010; Lilleri et al., 2008). However, these protocols are difficult to standardize and often laborious to adapt to GMP conditions. Furthermore, previous works have defined CD4+ and/or CD8+ T-cell responses to whole viral lysates, virally infected cells, recombinant proteins, and various HLA-restricted viral peptides.

The majority of studies have focused on the 65 kDa matrix phosphoprotein (pp65, also known as glycoprotein 64 and UL83) and the immediate-early protein 1 (IE1) of CMV as immunodominant targets of CMV-specific T-cell responses (Solache et al., 1999; Elkington et al., 2003; Sylwester et al., 2005). Regarding the induction of antiviral T-cell responses, pp65 has been recognized as a source of immunodominant epitopes that stimulate both CTLs and T helper cells. Most pp65-specific T cells predominantly produce effector cytokines like interferon-gamma (IFN-γ), interleukin-2 (IL-2) and tumor necrosis factor-alpha (TNF-α). The secretion of these cytokines is used for the detection and enrichment of antiviral T cells (Rauser et al., 2004). HLA class I-restricted peptides derived from CMV pp65 protein (e.g., the HLA-A^*^0201-restricted CMVpp65_495−503_ peptide) are known to be potent inducers of CTLs (Oelke et al., 2003; Paine et al., 2007). Because the known peptide epitopes are restricted to certain HLA alleles, the use of HLA-restricted peptides cannot exploit the full range of HLA diversity present in the patient. Furthermore, the use of HLA class I immunogenic peptides mainly leads to the generation of CD8+ T cells, resulting in the generation of an immune response restricted to cytotoxic T cells.

### Adoptive T-cell therapy for EBV infection

Adoptive T-cell therapy using EBV-CTLs has been successfully employed for prophylaxis and treatment of PTLD in high-risk patients (Haque et al., 2007; Heslop et al., 2010; Shen et al., 2011). EBV-transformed B-lymphoblastoid cell lines (B-LCLs) are established as antigen-presenting cells (APCs) for the generation of EBV-specific T cells. Following this approach, Tanzina Haque and colleagues (University of Edinburgh, UK) established and used a bank of frozen EBV-specific CTLs generated from the peripheral blood of Scottish blood donors to treat patients with progressive PTLD with CTLs selected on the basis of the best HLA-matches between the CTL donor and PTLD patient (Wilkie et al., 2004; Haque et al., 2007). In this multicenter clinical phase II trial, CTLs showed high efficacy varying according to the degree of HLA-match (at least 3/6) and did not induce any GvHD. Haque and colleagues demonstrated that the transfer of partially HLA-matched EBV-CTLs grown from healthy donors by repetitive antigenic stimulation is safe and results in tumor regression in about 60% of PTLD patients unresponsive to at least one prior treatment (Haque et al., 2007, 2010). In a study by Doubrovina et al., 49 HSCT patients with biopsy-proven EBV-lymphoproliferative disease (EBV-LPD) were treated with either HLA-compatible DLIs or HLA-compatible or HLA-disparate EBV-specific CTLs (Doubrovina et al., 2012). Acute GvHD was observed in 17% of all DLI recipients but in no EBV-CTL recipients. The data further supports the findings of Haque et al. indicating that EBV-CTLs from healthy, partially HLA-matched third-party donors provide an easily accessible source of effector T cells for the treatment of EBV-associated PTLD (Bollard et al., 2012; Doubrovina et al., 2012).

Nevertheless, the use of EBV-transformed cells as APCs to generate EBV-specific T cells for clinical use has three major limitations: (1) The manufacturing process for the B-LCL-based generation of sufficient numbers of EBV-CTLs for clinical use takes approximately 3 months (half for the generation of B-LCLs and half for T-cell expansion). Consequently, the production of EBV-CTLs for the individual patient with PTLD required diligent identification of patients at risk. Furthermore, the process is difficult to standardize and poses a potential biohazard due to the presence of live viruses. (2) So far, EBV-CTLs have mostly been manufactured from autologous peripheral blood mononuclear cells by repetitive *in vitro* stimulation with EBV antigens presented by APCs. In children, the lack of EBV infection prior to organ transplantation is an additional obstacle to the generation of sufficient numbers of EBV-CTLs. (3) The coverage of latency types is incomplete. Understanding the latency types of EBV is important for the effective design of adoptive T-cell strategies. While EBV-transformed B-LCLs express viral antigens representing latency type III (10 viral proteins), these cells might not be useful for generating specific T cells that effectively target late PTLDs expressing latency type II [only three viral proteins EBV nuclear antigen-1 (EBNA-1), late membrane proteins (LMP) LMP1 and LMP2 expressed] or latency type I tumors (e.g., Burkitt's lymphoma) [see review in (Bollard et al., 2012)]. The antigenic specificity of T cells generated by this method is further limited by the set of EBV proteins available in the B-LCLs—they contain mostly proteins from the early replication cycle (e.g., EBNA1-3) and no lytic proteins like BZLF. Unfortunately, EBNA-1, which is expressed in all three latency types, is poorly immunogenic (Thorley-Lawson and Allday, 2008). This was confirmed in studies analyzing the frequency of EBV-specific memory T cells in response to three commercially available peptide pools (EBNA-1, BZLF1, and LMP2A) in 195 healthy EBV-seropositive blood and platelet donors. It has been found that T-cell populations against the BZLF1-derived peptide pool were the most frequent in seropositive donors, as reflected by a high number of responders: 112 (57%) vs. 90 (49%) for EBNA-1 and 64 (33%) for LMP2A (Sukdolak et al., submitted).

### Adoptive T-cell therapy for ADV infection

Feuchtinger et al. clearly demonstrated that children with ADV-associated mortality had no ADV-specific T cells, whereas patients who cleared ADV infection had normal frequencies of antiviral T cells (Feuchtinger et al., 2006). Since an increased risk of adenoviral infection in immunocompromised patients has been shown to correlate with low numbers of T cells, efforts have been made over the past years to identify immunogenic ADV-derived epitopes. As of now, the 53 known human serotypes (ADV1 to ADV53) are classified into seven species (A to G). The most prevalent serotypes in transplant patients are ADV1, 2, 4, 5, 41, and 31. Hexon, the major capsid protein, serves as the immunodominant target antigen across the different serotypes of ADV. A few hexon-derived CD8^+^ T-cell epitopes for ADV species C have been identified and, for the most of them their clinical relevance remains unclear. This makes diagnosis and treatment very challenging. These epitopes are highly conserved, suggesting that ADV-specific T cells can cross-react with ADV serotypes and may therefore provide protection against a wide range of ADV strains (Zandvliet et al., 2010). Feuchtinger et al. tested the specific T-cell response to both hexon protein and whole ADV in HSCT donors and found that 10.5% of donors had a detectable T-cell response to whole ADV but no response to hexon protein, and 17% of donors had no detectable T-cell response to ADV (Feuchtinger et al., 2008). Zandvliet et al. were able to detect specific CD8^+^ T cells in 6/16 healthy donors after stimulation with 15-mer hexon peptide pool, while stimulation with peptides corresponding to known CD8^+^ hexon epitopes induced responses in 3/16 donors (Zandvliet et al., 2010). These studies clearly indicate the need to identify more immunodominant ADV epitopes.

## Strategies for isolation of antigen-specific T cells for adoptive T-cell therapy

Direct selection of virus-specific T cells without long-term *ex vivo* stimulation and manipulation is an attractive way to generate clinical-grade antiviral T cells. The two main approaches are separation by the use of cytokine secretion assays [e.g., interferon-gamma (IFN-γ) secretion assay (Rauser et al., 2004; Feuchtinger et al., 2008, 2010; Mackinnon et al., 2008; Moosmann et al., 2010; Peggs et al., 2011)] and isolation by the use of peptide-MHC (pMHC) multimers (Cobbold et al., 2005; Yao et al., 2008; Casalegno-Garduno et al., 2010; Schmitt et al., 2011). Direct isolation of antigen-specific T-cells by stimulation with antigenic peptides, proteins, or peptide-pools followed by cytokine capture and magnetic isolation is a rapid method of producing antiviral T-cells according to GMP guidelines (Rauser et al., 2004). It avoids safety and regulatory issues associated with prolonged T-cell culture and potential viral biohazards. Cytokine secretion assays using recombinant proteins or synthetic peptide pools consisting of overlapping peptides spanning an entire immunodominant protein are not restricted by HLA variations, and they enable the generation of CD4+ and/or CD8+ T-cell responses to multiple epitopes (Rauser et al., 2004). In the case of CMV 2, EBV 3, and ADV 1, GMP-grade peptide pools covering the viral proteins pp65 and IE-1 (CMV); LMP-2A, EBNA-1, and BZLF-1 (EBV); and the hexon (ADV) are now available for the generation of clinical-grade antiviral CD4+ and CD8+ T cells, irrespective of the HLA-type. It is known that specific CD4+ T-cell help is required to elicit and promote an efficient CD8+-restricted CTL response to viral antigens. CD4+ T cells secrete various cytokines to regulate and coordinate the function of T cells and other immune cells. They are also known to be the most effective cell population in clearing infections, such as ADV (Feuchtinger et al., 2006). Unfortunately, compared to the isolation of T cells by pMHC multimer technologies the purity is lower and alloreactivity of T cells might result in undesirable immune responses especially in HLA-mismatched or haploidentical settings. Nevertheless, clinical trials showed no increase in GvHD or graft rejection after adoptive immunotherapy using IFN-γ-isolated antiviral T cells (Feuchtinger et al., 2006; Peggs et al., 2011; Doubrovina et al., 2012).

The pMHC multimer technology requires knowledge of immunodominant HLA-restricted peptide epitopes and enables the isolation of either antigen-specific CD8+ T cells (pMHC class I multimers) or CD4+ T cells (pMHC class II multimers) of high purity. It is still difficult to generate the respective multimers needed for CD4+ T-cell isolation. Cobbold and colleagues, the first investigators to use tetramer-purified CMV-specific CD8+ T cells for adoptive transfer, were able to detect functionally active antiviral T cells within 10 days (Cobbold et al., 2005). In a study by Uhlin et al., tetramers corresponding to two EBV antigens were used to treat a patient suffering from PTLD after cord blood (CB) transplantation (Uhlin et al., 2010). Reversible pMHC multimers (streptamers, histamers), the latest generation of pMHC multimer technologies, were developed in order to isolate antigen-specific T cells without altering their functional status (Knabel et al., 2002; Tischer et al., 2012) and are already used clinical applications (Schmitt et al., 2011). Although the results are promising, this technology is limited to those donors who express an HLA allele with known viral epitopes and have sufficient numbers of memory T cells present in the peripheral blood.

T cells for adoptive immunotherapy could potentially be isolated from a T lymphocyte pool phenotypically identified as CD45RA+ CD62L+ naïve (N), CD45RO+ CD62L+ central memory (TCM), and CD62L- effector memory (TEM) T-cells subsets. These cells differ in phenotype, function, and homing (Sallusto et al., 2004). Recently it was shown, that although TEM have proliferative potential *in vitro*, these cells fail to survive in primates *in vivo* (Berger et al., 2008). These results most likely have implications for the types of T cells that should be selected for adoptive transfer.

## Do we need third-party T-cell donors?

The first clinical trials showed that T cells generated by the above-described procedures can be successfully used to treat viral infection, reactivation, or virus-induced malignancies after stem cell and SOT. It was also shown that adoptive immunotherapy with donor-derived virus-specific CTLs generated *in vitro* can effectively prevent the clinical manifestation of these viruses with no acute toxicity or increased risk of GvHD. In allogeneic stem cell transplantation, seropositive stem cell donors can usually serve as T cell donors and are available for T-cell donation. However, some seropositive donors may not consent, may be unavailable to provide T cells, or may not have enough antiviral memory T cells in their blood despite seropositivity. Recent studies have also shown that granulocyte colony-stimulating factor (G-CSF) mobilization has a long-term negative effect on the functional activity of T cells (Franzke et al., 2003; Toh et al., 2009). Bunse et al. (in preparation), suggesting that antiviral memory T cells from stem cell donors might not be the best source. Furthermore, delayed hematologic engraftment and immune reconstitution are a major problem in patients receiving CB transplants. These problems may be attributable to a low stem cell dose, small numbers of transferred T cells, the absence of memory T cells within the CB grafts, or the hyporesponsiveness of CB APCs. Therefore, these patients are at high risk of developing viral complications as are patients receiving transplants from seronegative donors or SOT patients receiving organ grafts from deceased donors. CMV-seropositive immunocompromised patients (R+) with transplants from seronegative donors (D−) were shown to have a high risk of CMV disease (Zhou et al., 2009; Ugarte-Torres et al., 2011). It was reported that CMV reactivation occurs in 96% of D+R+ patients but in less than 50% of D+R– patients (Lilleri et al., 2008; Borchers et al., 2011). Therefore, adoptive transfer of virus-specific CTL is not a viable option for high-risk patients (R+) with seronegative donors (D−).

Under these conditions, partially HLA-matched virus-specific T cells from healthy seropositive individuals could be a successful alternative and could play a significant role in the prevention and treatment of viral infections in transplant recipients. Studies on the use of HLA-matched T-cells from third-party donors for the treatment of stem cell and organ recipients are currently in progress.

The third-party approach was first successfully tested in SOT and HSCT patients with EBV-associated PTLD at the University of Edinburgh (Haque et al., 2002, 2007; Wilkie et al., 2004). As mentioned in chapter 4.2 Haque and colleagues showed that partially HLA-matched EBV-specific T cells (at least 3/6) produce a 65% response rate and a 42% complete response rate in PTLD patients after SOT, indicating that the transferred EBV-specific T cells were highly effective and did not induce any GvHD (Haque et al., 2007, 2010). Later studies (Barker et al., 2010; Uhlin et al., 2010; Doubrovina et al., 2012) including CB transplant patients confirmed these results. O'Reilly and colleagues used partially HLA-matched EBV-specific T cells to treat EBV lymphoproliferative disease in allogeneic HSCT recipients and achieved complete or partial remission in 68% (Barker et al., 2010; Doubrovina et al., 2012).

The effectiveness of third-party donor-derived T cells in treating CMV and ADV is now being investigated in various clinical trials (e.g., in Memorial Sloan-Kettering Cancer Center's phase II trial on the treatment of CMV). Feuchtinger et al. reported their results obtained with T cells from two third-party, partially HLA-matched, unrelated CMV-specific T-cell donors to treat CB transplanted patients (Feuchtinger et al., 2010). The cells were rapidly isolated from the donors using an IFN-γ cytokine secretion assay after brief stimulation of peripheral blood mononuclear cells with CMV pp65. *In vivo* expansion of CMV-specific T cells and clearance of CMV infection was observed in one patient (Schottker et al., 2008; Feuchtinger et al., 2010). Third-party virus-specific T cells directed against ADV were also shown to be effective for the eradication of ADV (Qasim et al., 2011). Rooney and Leen recently investigated the use of banks of third-party T cells specific for CMV, EBV, and ADV in HSCT patients and observed a high (>70%) response rate to all three viruses, even in case of only one HLA allele match between the CTL line and the recipient (Gerdemann et al., 2012).

### The allogeneic T-cell donor registry

First results using T cells from partially HLA-matched third-party donors are promising. The data indicate that allogeneic T-cell therapy is an attractive option for patients suffering from viral infections after allogeneic HSCT or organ transplantation. Therefore, we hypothesize that a registry of HLA-typed allogeneic T-cell donors typed for virus-specific T cells would enable rapid availability of T cells for adoptive immunotherapy of virus-associated diseases in transplant recipients without an adequate T-cell donor. This registry might provide a stand-alone off-the-shelf product.

To gain more insight into virus-specific memory T-cell pools in healthy donors and to identify the most efficient antigens for adoptive immunotherapy, we determined the frequencies of virus-specific memory T cells in healthy donors. To date, T-cell frequencies have been determined in more than 300 HLA high-resolution typed donors at Hannover Medical School's Institute for Transfusion Medicine by INF-γ enzyme-linked immunospot (ELISpot) assay and flow cytometry using pMHC multimers (Sukdolak et al., submitted). Using these well-established methods of T-cell monitoring (Cox et al., 2006; Hadrup and Schumacher, 2010), we assessed the frequencies of T cells against GMP-quality peptides and peptide pools derived from viral proteins known to be immunodominant or subdominant. For example, phosphoprotein 65 (pp65) and immediate early (IE)−1 were used for CMV (Wills et al., 1996), BZLF1, nuclear antigen (EBNA)−1 and latent membrane protein 2A (LMP2A) for EBV (Houssaint et al., 2001), and hexon, the major capsid protein of ADV, for ADV (Leen et al., 2008). For optimal T-cell help and cytotoxic response, the T-cell population should consist of CD4+ and CD8+ virus-specific T cells. For high efficiency, these cells should also target various viral epitopes. For each virus studied, we identified at least 61% potential CTL donors with highly significant differences in frequencies of T cells against each of the six viral antigens tested: pp65 and IE-1 (CMV), BZLF1, LMP2A, and EBNA1 (EBV), and hexon (ADV).

All CMV-seropositive donors were reactive to the CMV pp65 peptide pool, whereas only 79% reacted with IE-1. One hundred and seventy three of the EBV-seropositive donors had antigen-specific T cells that reacted with at least one of three EBV peptide pools, showing highest frequencies for BZLF1, and 73% of the ADV-seropositive donors reacted with the hexon peptide pool. Interestingly we found that in short-term *in vitro* peptide stimulation assays for ADV and EBV a donor response to a certain peptide may not be determined on day 0. Peptide-specific T cells were detected by multimer staining, but overall frequencies were lower than those obtained for the corresponding peptide pools. The results of our study demonstrate that, depending on the antigen used, no antiviral T cells can be detected in approximately one-third of donors despite seropositivity, and that serological testing for CMV by the standard ELISA technique gives false-positive results in approximately 10% of donors. It is important to remember that GvHD remains a dreaded side effect and there is a particularly risk of alloreactivity, especially in partially-HLA-matched settings (Amir et al., 2010; D'Orsogna et al., 2010; Qasim et al., 2011). Therefore, we developed a modified granzyme B ELISpot assay to detect T-cell specificity and alloreactivity against patient cells and used it to test T-cell effector function against unloaded PBMCs, (HLA class I-negative) K562 cells and “patient unloaded and antigen-loaded PBMCs.” This method can also be used to identify the best HLA-matched allogeneic antiviral CTL donor. The HLA-types of CTLs with the highest specific killing of “patient antigen-loaded PBMCs” were identified and considered in partially HLA-matched allogeneic T-cell therapy.

The results were used to establish a registry of potential T-cell donors (allogeneic T-cell donor registry, alloTCDR) with highly virus-specific T-cell precursors. The alloTCDR database will document the donors' HLA-type (class I and II high resolution), virus serology (ADV, CMV, and EBV), virus-specific T-cell frequencies, best T-cell detection method, and results of functional and alloreactivity assays. This registry of HLA-typed allogeneic T-cell donors profiled for virus-specific T cells will ensure the rapid availability of T cells for adoptive immunotherapy of virus-associated diseases in transplant recipients without an adequate T-cell donor.

## Summary

Antigen-specific T cells can be effectively used in the treatment of viral infection or reactivation after stem cell and SOT. So far most studies did not show significant increase in the development of acute toxicity or increased risk of GvHD following T-cell transfer. Unfortunately, for patients receiving an allogeneic CB transplant, a transplant from a virus-naïve donor or a transplant from a cadaveric donor no T-cell donor will be available. Furthermore, it was shown that in some cases no antiviral memory T cells are present in the donor despite seropositivity, and that G-CSF treatment has a negative effect on antiviral cell function. Third-party partially HLA-matched virus-specific T cells from healthy seropositive individuals may be an option, which can be successfully employed under these circumstances. In future, we will extend the typing and profiling of potential third-party donors to include the T-cell frequencies of other viruses, such as polyoma virus BK, human herpesvirus 6, and invasive fungal pathogens such as *Aspergillus*. The registry of unrelated HLA-typed allogeneic T-cell donors profiled for virus-specific T cells will ensure the rapid availability of T cells for adoptive immunotherapy of pathogen-associated diseases in transplant recipients.

### Conflict of interest statement

The authors declare that the research was conducted in the absence of any commercial or financial relationships that could be construed as a potential conflict of interest.
